# Cofilin overactivation improves hippocampus-dependent short-term memory

**DOI:** 10.3389/fnbeh.2023.1243524

**Published:** 2023-08-10

**Authors:** Frank Raven, Iris W. Riemersma, Martha F. Olthuis, Ieva Rybakovaite, Elroy L. Meijer, Peter Meerlo, Eddy A. Van der Zee, Robbert Havekes

**Affiliations:** Neurobiology Expertise Group, Groningen Institute for Evolutionary Life Sciences (GELIFES), University of Groningen, Groningen, Netherlands

**Keywords:** cofilin, hippocampus, memory, synaptic plasticity, AMPA receptor

## Abstract

Many living organisms of the animal kingdom have the fundamental ability to form and retrieve memories. Most information is initially stored as short-term memory, which is then converted to a more stable long-term memory through a process called memory consolidation. At the neuronal level, synaptic plasticity is crucial for memory storage. It includes the formation of new spines, as well as the modification of existing spines, thereby tuning and shaping synaptic efficacy. Cofilin critically contributes to memory processes as upon activation, it regulates the shape of dendritic spines by targeting actin filaments. We previously found that prolonged activation of cofilin in hippocampal neurons attenuated the formation of long-term object-location memories. Because the modification of spine shape and structure is also essential for short-term memory formation, we determined whether overactivation of hippocampal cofilin also influences the formation of short-term memories. To this end, mice were either injected with an adeno-associated virus expressing catalytically active cofilin, or an eGFP control, in the hippocampus. We show for the first time that cofilin overactivation improves short-term memory formation in the object-location memory task, without affecting anxiety-like behavior. Surprisingly, we found no effect of cofilin overactivation on AMPA receptor expression levels. Altogether, while cofilin overactivation might negatively impact the formation of long-lasting memories, it may benefit short-term plasticity.

## 1. Introduction

The capacity to form and retrieve memories enables an organism to adapt to an ever-changing environment, and therefore is necessary for survival (Bruel-Jungerman et al., 2007; Rasch and Born, 2013). Even though many types of memory require complex interactions between multiple brain structures, one brain structure highly important for declarative memories, including spatial and episodic memory processing, is the hippocampus. Earliest work from Scoville and Milner (1957) indicated that the hippocampus is crucial for the acquisition and retention of memories (reviewed in: Squire and Wixted, 2011; Kandel et al., 2014). Numerous studies examining the role of the hippocampus in the different aspects of learning and memory, underscored the importance of this brain region in the formation of both short-term and long-term spatial memories (Havekes and Abel, 2009).

Within the hippocampus, memory formation requires both synaptic, as well as structural plasticity (Yuste and Bonhoeffer, 2001; De Roo et al., 2008), with the latter emphasizing changes in synaptic morphology. These phenomena take place at the level of the synapse, and involve dendritic spines, which are specialized actin-rich compartments that contain many neurotransmitter receptors and several other organelles (Cajal, 1894; Matus et al., 1982; Dunaevsky et al., 1999; Matus, 2000; Sorra and Harris, 2000; Kasai et al., 2010). Changes in the shape of a spine involves remodeling of actin filaments by balancing assembly and disassembly of the filaments (Yuste and Bonhoeffer, 2001). One important factor in the regulation of actin dynamics in dendritic spines, is the protein cofilin (Bamburg and Bray, 1987; Moriyama et al., 1990; Racz and Weinberg, 2006; Rex et al., 2009). Cofilin depolymerizes actin filaments, however, when it becomes phosphorylated on Ser3, it becomes inactive and therefore loses its destabilizing properties (Bamburg and Wiggan, 2002; Van Troys et al., 2008; Bernstein and Bamburg, 2010). Hence, the balance between active and inactive cofilin regulates spine dynamics (Raven et al., 2017). Interestingly, the level of (active) cofilin rises directly after the induction of long-term potentiation (LTP)—a cellular model to investigate memory based on the electrical properties of a neuron (Bliss and Lømo, 1973)—indicating increased plasticity. Shortly after LTP induction, levels of cofilin return to baseline, implying a period of spine stability (Chen et al., 2007). After this period, during memory consolidation, levels of phosphorylated (inactive) cofilin are increased in the hippocampus (Fukazawa et al., 2003; Chen et al., 2007; Fedulov et al., 2007; Rex et al., 2009; Suzuki et al., 2011). However, it is unknown whether high levels of active cofilin might be beneficial for other memory types, such as short-term memory.

Cofilin is also involved in α-amino-3-hydroxy-5-methyl-4-isoxazolepropionic acid (AMPA) glutamate receptor trafficking. The AMPA receptors are highly expressed on postsynaptic dendritic spines (Beneyto and Meador-Woodruff, 2004) and facilitate synaptic transmission, which is essential for synaptic plasticity and learning and memory (Matsuzaki et al., 2001; Sanderson et al., 2008). Furthermore, AMPA receptors consist of four subunits. Phosphorylation of the GluR1 subunit at specific sites (e.g., serine site 831) is crucial for the retention of spatial memories and synaptic plasticity (Lee et al., 2003). In addition, during LTP, cofilin moderates AMPA receptor trafficking to the postsynaptic surface, which was surprisingly not directly coupled to changes in spine morphology (Gu et al., 2010). Therefore, the regulation of cofilin is crucial for synaptic plasticity memory, also via the regulation of AMPA receptors. However, it is unclear whether constitutive activation of cofilin alters AMPA receptor phosphorylation levels.

Altogether, these data suggest that elevating cofilin levels would negatively affect spine stability and long-term memory consolidation. Indeed, recent data showed that injecting an adeno-associated virus (AAV), leading to the expression of a dominant active form of cofilin specifically in the hippocampus, impaired long-term spatial memory (Havekes et al., 2016b). Despite the accumulating evidence that (active) cofilin is elevated directly after LTP induction and mediates AMPA receptor trafficking—which are both important for short-term memory—no studies have examined whether cofilin overactivation specifically in the hippocampus affects short-term spatial memory. Therefore, in the current study we investigated the effect of cofilin overactivation, specifically in the hippocampus, on short-term memory and related plasticity.

## 2. Materials and methods

### 2.1. Subjects

For this study, male and female C57BL/6 mice were obtained from Charles River Laboratories. The mice were housed on a 12/12 hr light/dark cycle with lights on at 10 a.m. Food and water were available *ad libitum*. Behavioral experiments started 4 weeks after surgery, at which point the viral cofilin expression levels were high enough to outcompete endogenous cofilin levels (Havekes et al., 2016b). All behavioral experiments were conducted at the beginning of the light phase, and different batches of mice were used for studying object-location memory and anxiety-like behavior. At 5–6 months of age mice were sacrificed by transcardial perfusion or cervical dislocation, and brains were collected for further immunohistochemical or biochemical analyses (Havekes et al., 2012). All experiments were approved by the national Central Authority for Scientific Procedures on Animals (CCD) and the Institutional Animal Care and Use Committee (IVD) of the University of Groningen.

### 2.2. Viral constructs and surgeries

Adeno-associated viruses pAAV9-CaMKIIα0.4-Cofilin^*S*3*A*^-HA and pAAV9-CaMKIIα0.4-eGFP were used to overexpress active cofilin (cofilin^*S*3*A*^) or enhanced Green Fluorescent Protein (eGFP, control), respectively, in the mouse hippocampus (Havekes et al., 2016a,b). The CaMKIIα (Calcium/calmodulin-dependent protein kinase II alpha) promoter was used to restrict the expression of the viruses to excitatory neurons (Havekes et al., 2016a). To discriminate endogenous cofilin from the viral exogenous cofilin, an HA-tag was added to the viral construct of cofilin^*S*3*A*^ (Havekes et al., 2016b). The viral vectors were produced at the viral core of the University of Pennsylvania.

Before the start of the surgery, mice were anaesthetized with isoflurane and received Finadyne (50 μg/kg i.p.) as an analgesic. Throughout the surgery, the animals were placed on a heating pad to maintain body temperature. Artificial tears (Duratears Z; Alcon) were used to protect the eyes from drying out. Mice were positioned on a stereotax, and after making a small incision, Ketac™ Conditioner Polyacrylic Acid (3M) was used to identify Bregma, allowing the determination of the coordinates for hippocampal injection. The coordinates of the bilateral injections were; A/P −2.0 mm, L/M ± 1.5 mm and D/V −1.5 mm below bregma. Holes were drilled at the place of the injections using a microdrill (Foredom). A microsyringe pump (UMP3; WPI) connected to a mouse stereotax and controller (Micro4; WPI) was used to control the speed of injection (0.2 μL per min.). Mice were injected with 1 μL Cofilin^*S*3*A*^ virus per hippocampus (titer: 2.52 × 10^13^ genome copy numbers). Control animals were injected with eGFP virus, 0.25 μL per hippocampus (titer: 6.27 × 10^13^ genome copy numbers). The holes in the mouse’s skull were sealed with bone wax (Sharpoint bone wax; Surgical Specialties Corporation, PA, USA). The incision was sutured (Supramid USP 4/0 EP 1.5; SMI AG) and the mouse was removed from the stereotax and placed in a cage on a heating pad until it regained conscious.

### 2.3. Elevated plus maze

An elevated-plus maze was used to investigate whether cofilin overactivation affects anxiety-like behavior. More time spent in the closed arms compared with the open arms is an indication of increased anxiety-like behavior (Pellow et al., 1985; Walf and Frye, 2007). The arms of the maze are 32.5 cm long and 5 cm wide, elevated 75 cm off the ground. The maze consists of two open arms and two arms enclosed by 16 cm high walls. The mouse was placed in the center of the maze and was allowed to freely explore for 8 min. Videos were recorded and analyzed with the program Media Recorder (Noldus Information Technology, Wageningen, The Netherlands). Time spent in open arms was scored which was defined as four limbs passing the threshold of an arm.

### 2.4. Object-location memory

Object-location memory (OLM) test was used to examine spatial memories. The OLM task relies on the rodent’s innate preference for spatial novelty, and previous work has shown that the consolidation of object-location memories are dependent on the hippocampus (Oliveira et al., 2010; Havekes et al., 2014). Before the start of the experiment, mice were handled for 2 min for five consecutive days in the testing room. On the training day, mice were first habituated to the test box. Mice were allowed to freely explore the rectangular test box (40 cm × 30 cm × 30 cm) with a visual cue placed on one of the test box walls, in absence of objects for 10 min. Subsequently, three training sessions were performed in which mice were placed in the same box containing three similar objects, and allowed to explore for 10 min. One hour later, the mouse was placed in the same test box containing three objects, of which one was moved to a novel location, and allowed to explore for 10 min. The box was cleaned with 70% alcohol and the objects were cleaned with 10% alcohol between sessions. Each training or test session was recorded, and the exploration time of each object was scored blindly by the experimenter using Observer (Noldus Information Technology, Wageningen, The Netherlands). Exploration was defined as being within 1 cm radius of the object with the nose directed toward the object and/or touching the object. Leaning or climbing the object was not considered exploration. Increased amount of time spent exploring the displaced object indicates object-location memory.

### 2.5. Immunohistochemistry

Mice were perfused by transcardial perfusion and brains were collected for immunohistochemistry to determine whether the virus was injected in the correct location. Mice were first perfused with 0.9% NaCl and 2 U/mL heparin followed by fixation solution containing 4% PFA in 0.1M PB, after which the brains were post fixated in the fixative for 24 h at 4°C. After dehydration with 30% sucrose, brains were frozen and sliced in 30 μm thick coronal sections in a cryostat (Leica LM 3050) at −14°C.

Staining for the cofilin^*S*3*A*^ HA-tag was performed as described previously (Havekes et al., 2016b). In short, sections were rinsed with TBS, blocked TBS with 3% BSA and 0.1% Triton™ X-100. Subsequently, sections were incubated with the following antibodies: anti-HA-tag (1:200, Roche, RRID:AB_390918), anti-Mouse Anti-Glial Fibrillary Acidic Protein (GFAP; 1:20000, Sigma-Aldrich, RRID:AB_477010), anti-Iba-1 (1:2500, Wako, RRID:AB_839504), and corresponding Alexa fluor-conjugated secondary antibodies (1:500, Invitrogen, AB_162542, AB_162543, AB_2535794). Fluorescent images were taken using a confocal microscope.

### 2.6. Western blot

Animals were sacrificed by cervical dislocation and hippocampi were collected and homogenized in a cold homogenization buffer (Tris 50 mM, Sodium Deoxycholate 1%, NaF 50 mM, Sodium Vanadate 20 μM, EDTA 20 μM, Beta Glycerophosphate 40 μM) with Roche Tablets (catalogue number:11836170001) using a Tissuelyser Adapter Set (Qiagen, Germany) (Havekes et al., 2016b). Bradford analyses was used to determine the protein concentration. Samples were loaded in pre-cast Bolt™ 4–12% Bis-Tris Plus (Invitrogen, USA), and after electrophoresis blotted with iBlot™ Gel Transfer System (Invitrogen, USA). Then, the membranes were blocked for 1 h and incubated overnight with either one of the following primary antibodies: anti-p-cofilin (1:750, Cell Signaling Technology, RRID:AB_2080597), anti-cofilin (1:2000, BD Biosciences, RRID:AB_399516), anti-GAPDH (1:3000, Thermo Fisher Scientific, RRID:AB_568547), anti-p-AMPA receptor ser831 (1:3000, Millipore, RRID:AB_1977218), anti-total AMPA receptor GluR1 (1:1000 Genetex, RRID:AB_11168026). Subsequently, membranes were incubated with the corresponding HRP-conjugated secondary antibodies for 2 h (goat-anti mouse, Santa Cruz Biotechnology RRID:AB_631736; goat-anti rabbit RRID:AB_2099233). GAPDH was used as loading control. Bands were visualized with Pierce™ ECL solution (ThermoFisher Scientific, USA) and Molecular Imager ChemiDoctm XRS System (Bio-Rad, USA).

### 2.7. Statistics

All analyses were conducted by an experimenter blind to treatment. Statistical differences in behavior between the cofilin^*S*3*A*^ mice and control eGFP mice were examined using a two-way ANOVA. Sex was included as an independent variable. A repeated-measures ANOVA was used to investigate exploration times during the training phase of the OLM, in which training session was the within-subject factor, and sex and group were the between-subject factors. Differences in protein levels between control and cofilin^*S*3*A*^ mice were calculated with a one-way ANOVA. The Tukey procedure was used for *post hoc* analysis when necessary. A non-parametric Independent-Samples Mann-Whitney U Test was used in case variance was not homogeneously distributed between groups. Data are presented as the mean, the area (band) around the mean represents the SEM, the smoothed density curve (bean) indicates the full data distribution, dots show the individual data points (pirateplot, yarrr package, Nathaniel D. Phillips). Data were analyzed using the SPSS 24.0 software (IBM Corp., Armonk, NY, USA). Differences were considered statistically significant when *p* < 0.05.

## 3. Results

### 3.1. Cofilin^*S*3*A*^ is expressed in hippocampal excitatory neurons

**FIGURE 1:**
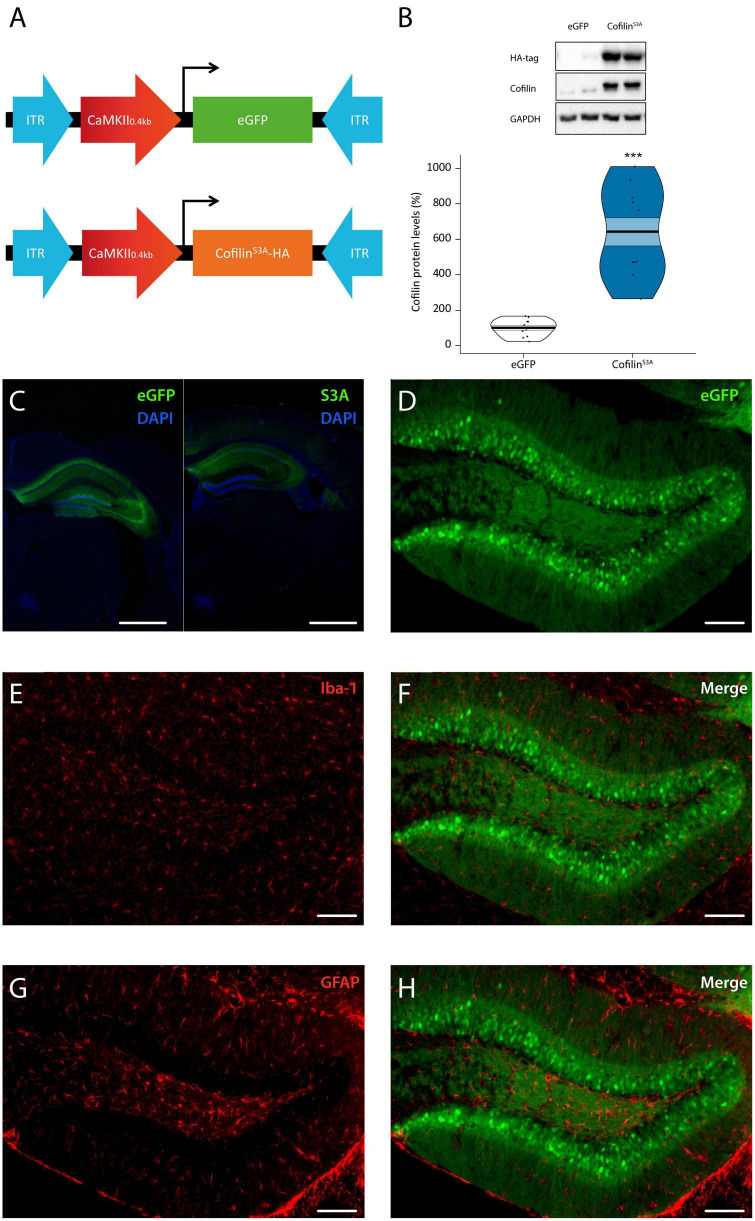
Cofilin overactivation in the hippocampus. **(A)** Mice were bilaterally injected with pAAV9-CaMKIIa0.4-eGFP or pAAV9-CaMKIIa0.4-cofilin^S3*A*^-HA to express either eGFP or the catalytically active version of cofilin (cofilin^S3A^) in hippocampal excitatory neurons. The active version of cofilin was created by replacing Serine 3 for Alanine. An HA-tag was used to discriminate between mutant and endogenous cofilin. **(B)** Western blot data showing that virally delivered cofilin^S3A^ protein levels increased cofilin activity by an approximate 6-fold compared to protein levels of control animals (test statistic_1, 19_ = 110.00, *p* < 0.001). An HA-tag antibody was used to detect the mutant active form of cofilin. A representative blot is depicted in which each band represents an individual animal. Data are presented as the mean, the area (band) around the mean shows the SEM, the smoothed density curve (bean) indicates the full data distribution, dots represent the individual data points. eGFP *n* = 11 (all males); cofilin^S3A^ *n* = 10 (all males). **(C,D)** Representative images showing that viral eGFP or cofilin^S3A^ expression was restricted to the hippocampus, excluded from microglia **(F)** and astrocytes **(H)** in the dentate gyrus of the hippocampus, indicated by a lack of co-labeling with iba-1 **(E)** and GFAP **(G)** expression, respectively. Scale bar, 1 mm **(C)**, 100 μm **(D–H)**. ***Indicates *p* < 0.001.

We first assessed whether expression of cofilin^*S*3*A*^ is limited to hippocampal neurons, and whether total cofilin levels were increased, as cofilin^*S*3*A*^ is also detected by the total cofilin antibody. An overview of the different AAVs used in this study is shown (Figure 1A). Western blot analyses of hippocampal lysates indicated that the cofilin^*S*3*A*^ was indeed detectable as indicated by the HA-bands (Figure 1B). In addition, the cofilin levels were increased by approximately a 6-fold compared to eGFP control (test statistic_1,19_ = 110.00, *p* < 0.001; Figure 1B). Furthermore, viral expression of cofilin^*S*3*A*^ did not alter p-cofilin levels, which was examined in a separate batch of animals (data not shown). We found that expression was restricted to the dorsal hippocampus and part of the ventral hippocampus (representative images are shown in Figure 1C, see Supplementary Figures 1A, B for dorsal and ventral images of the hippocampus, respectively). Subsequently, we wanted to verify that non-neuronal cells, such as microglia and astrocytes did not express the virus. Double labeling studies demonstrated that virally expressed neurons did not co-localize with microglia, as indicated by iba-1 positive cells (Figures 1D–F), or astrocytes, as indicated by GFAP positive neurons (Figures 1D, G, H). Together, these data show that we could successfully express cofilin^*S*3*A*^ selectively and robustly into hippocampal excitatory neurons.

### 3.2. Cofilin overactivation improves short-term object-location memory

**FIGURE 2:**
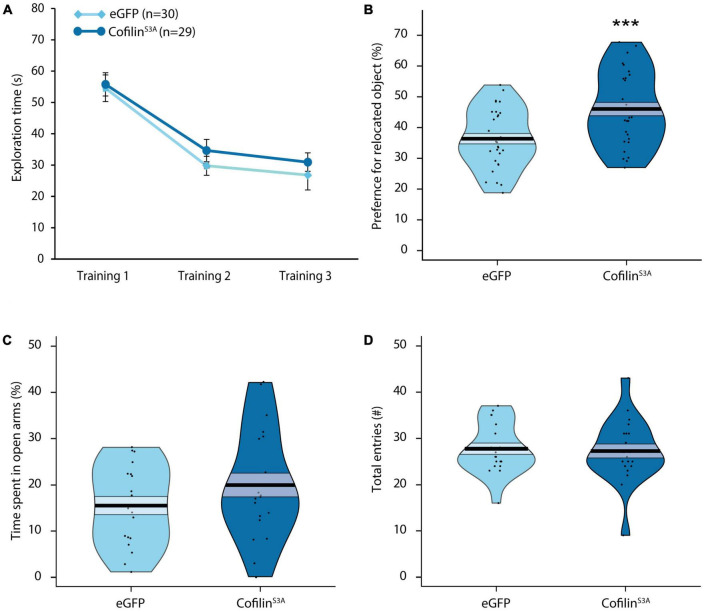
Cofilin overactivation in the hippocampus improves short-term memory and does not affect anxiety-related behavior. Mice either injected with cofilin^S3A^ or eGFP were trained for object-location memory and tested 1 hr after training. **(A)** There was a significant decrease in exploration time across the three training sessions (*p* < 0.001). In addition, there were no differences in total exploration time between the groups during the training (*p* = 0.832). **(B)** Mice injected with cofilin^S3A^ performed better than eGFP injected mice (*p* < 0.001). **(C)** Percentage of time spent in open arms of the elevated plus maze of mice either injected with cofilin^S3A^ or a control eGFP expressing virus. There were no differences in percentage of time spent in open arms (*p* = 0.166). **(D)** There was also no difference in the amount of entries made between cofilin^S3A^ and eGFP injected mice (*p* = 0.775). Data are presented as the mean, the area (band) around the mean represents the SEM, the smoothed density curve (bean) indicates the full data distribution, dots show the individual data points. **(A,B)**: eGFP, *n* = 30 (male: *n* = 14), cofilin^S3A^, *n* = 29 (male: *n* = 14). **(C,D)**: eGFP, *n* = 19 (male: *n* = 9), cofilin^S3A^, *n* = 20 (male: *n* = 9). ***Indicates *p* < 0.001.

As a next step, we investigated whether cofilin overactivation would have functional effects at the behavioral level. Because the consolidation of OLM requires the activation of the hippocampus (Oliveira et al., 2010; Florian et al., 2011), we used this task to examine the effect of hippocampal cofilin overactivation at the behavioral level. Even though previous work showed that cofilin overactivation decreased long-term OLM (Havekes et al., 2016b), we questioned how overexpression of constitutively active cofilin levels would impact short-term OLM. As expected, there was a significant decrease in total exploration time across the three training sessions (*F*_2,110_ = 60.406, *p* < 0.001; Figure 2A), which did not differ between groups (*F*_1,55_ = 0.045, *p* = 0.832) or sexes (*F*_1,55_ = 1.597, *p* = 0.212). There was also no significant three-way interaction effect between training session, group and sex (*F*_2,110_ = 0.840, *p* = 0.435). Neither was there a significant interaction between training session and group (*F*_2,110_ = 0.858, *p* = 0.427), nor an interaction between training session and sex (*F*_2,110_ = 0.117, *p* = 0.890). These analyses indicate that hippocampal cofilin^*S*3*A*^ expression does not affect exploratory activity during the training sessions of the OLM task (see Supplementary Figure 2 for OLM training results per sex).

As can be seen from Figure 2B, cofilin^*S*3*A*^ mice spent significantly more time exploring the relocated object relative to control animals (*F*_1,55_ = 11.683, *p* < 0.001) during the short-term memory test, 1 h after training. There was, however, a significant sex effect, in which males in general spent more time at the relocated object, independent of viral treatment (*F*_1,55_ = 4.676, *p* < 0.05). Furthermore, there was no significant interaction effect between group and sex (*F*_1,55_ = 0.414, *p* = 0.522). These data indicate that the injection of cofilin^*S*3*A*^ did not affect male and female mice differently. However, while the cofilin^*S*3*A*^ mice performed above chance level (t1, 28 = 5.674, *p* < 0.001; see Supplementary Figure 2 for male—female differences), upon closer inspection of the data we found that eGFP mice only showed a trend toward exploring the relocated object (t1, 29 = 1.741, *p* = 0.092). While male eGFP mice performed above chance level (t1, 13 = 2.679, *p* < 0.05), female eGFP expressing mice did not spend more time with the relocated object compared to chance level (t1, 15 = −0.284, *p* = 0.781; see Supplementary Figure 2B for graphs separated for males and females). In addition, total exploration during the test session did not differ between cofilin^*S*3*A*^ and eGFP mice (*F*_1,55_ = 1.273, *p* = 0.264), or sexes (*F*_1,55_ = 0.868, *p* = 0.356), nor was there an interaction effect between group and sex (*F*_1,55_ = 0.335, *p* = 0.565). Altogether, cofilin^*S*3*A*^ expression in the hippocampus improved short-term memory in the OLM task.

### 3.3. Cofilin overactivation does not affect anxiety behavior

Because a change in anxiety could affect the outcome in the OLM task, as a next step we assessed the impact of cofilin^*S*3*A*^ overexpression on exploratory and anxiety-like behavior using the elevated plus maze (Walf and Frye, 2007). Cofilin^*S*3*A*^ mice did not spend more time in the open arms compared to eGFP mice (*F*_1,35_ = 1.898, *p* = 0.177; Figure 2C). Furthermore, there was also no sex effect (*F*_1,35_ = 0.916, *p* = 0.345; also see Supplementary Figure 2C for results separated by sex), nor an interaction effect between group and sex (*F*_1,35_ = 0.396, *p* = 0.533; Figure 2C). Furthermore, mice expressing cofilin^*S*3*A*^ made a similar amount of entries compared to eGFP injected mice (*F*_1,35_ = 0.060, *p* = 0.808; Figure 2D). Although there was no interaction effect of group and sex (*F*_1,35_ = 0.410, *p* = 0.526), there was a significant sex effect in which males in general made more entries than female mice (*F*_1,35_ = 7.029, *p* < 0.05; see Supplementary Figure 2D). In summary, even though male mice made more entries than female mice, hippocampal cofilin^*S*3*A*^ overexpression did not affect anxiety-like behavior.

### 3.4. Cofilin overactivation affects memory probably in an AMPA receptor independent manner

**FIGURE 3:**
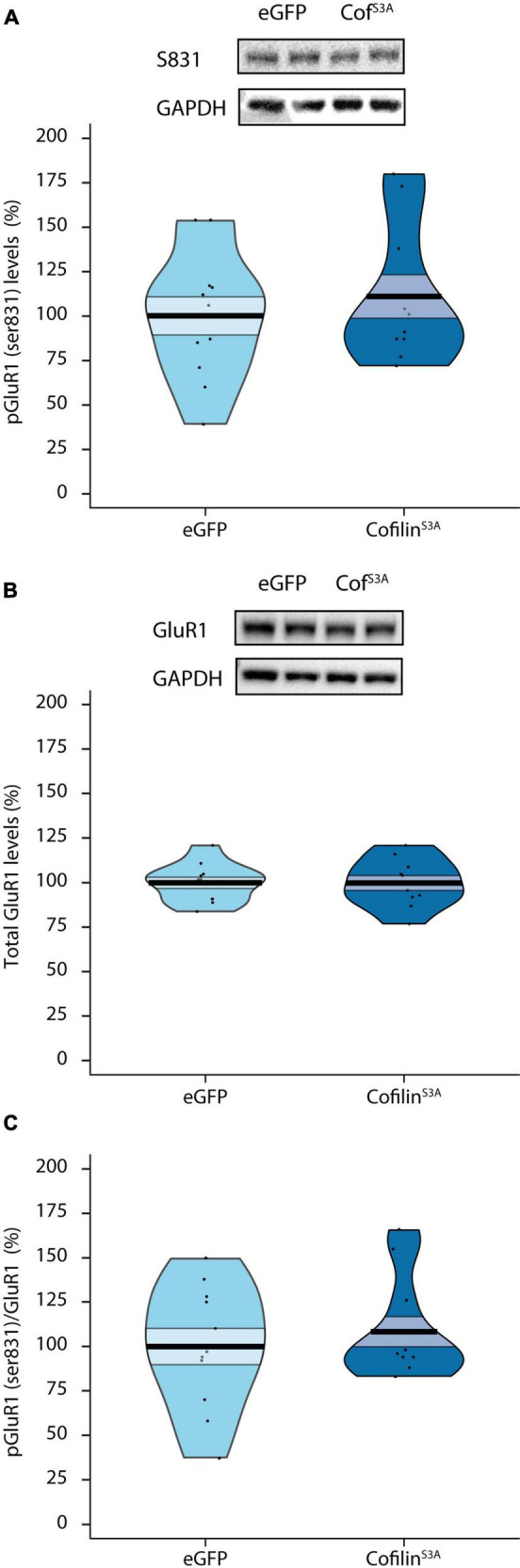
Cofilin overactivation does not affect phosphorylation or total levels of GluR1 AMPA receptors. Effects of cofilin overactivation on hippocampal phosphorylated and total GluR1 AMPA receptor levels. **(A)** Phosphorylated GluR1 (ser831) protein levels in lysated hippocampi from eGFP and Cofilin^S3A^ expressing mice (*p* = 0.514). **(B)** Total GluR1 levels did not differ between groups (*p* = 0.986). **(C)** Cofilin overactivation did not affect the pGluR1 (ser831):GluR1 ratio (*p* = 0.509). A representative blot is shown in which each band represents an individual animal. Data are presented as the mean, the area (band) around the mean represents the SEM, the smoothed density curve (bean) indicates the full data distribution, dots show the individual data points. Cof^S3A^: Cofilin^S3*A*^-expressing mice. eGFP, *n* = 11 (all males); cofilin^S3A^, *n* = 10 (all males).

Next, we examined effects of cofilin^*S*3*A*^ on the GluR1 subunit of the AMPA receptor, which is highly important for hippocampus-dependent short-term memory (Lee et al., 2003; Kessels and Malinow, 2009; Sanderson et al., 2010). Specifically, we investigated whether cofilin overactivation would affect the phosphorylation status of Serine 831 on the GluR1 AMPA receptor, as this site is found to be crucial for integration of the GluR1 AMPA receptor into the synaptic membrane and LTP via CaMKII (Banke et al., 2000; Esteban et al., 2003). Surprisingly, there was no difference in phosphorylation levels of GluR1 receptor at Serine 831 between cofilin^*S*3*A*^ and eGFP-injected animals (*F*_1,19_ = 0.442, *p* = 0.514; Figure 3A). In addition, total GluR1 levels did not differ between groups (*F*_1,19_ = 0.000, *p* = 0.986; Figure 3B). To see if cofilin overactivation affects the specific phosphorylation status compared to general GluR1 AMPA receptor levels, we calculated the ratio between GluR1 Serine 831 phosphorylation and total GluR1 levels. As can be seen in Figure 3C, cofilin overactivation did not affect the pGluR1 (ser831):GluR1 ratio (*F*_1,19_ = 0.453, *p* = 0.509). Hence, cofilin overactivation likely does not affect the phosphorylation or total levels of GluR1 AMPA receptor (see Supplementary Figure 3 for the original blots).

## 4. Discussion

Here, we examined the effect of cofilin overactivation specifically in hippocampal excitatory neurons on aspects of cognition and related synaptic plasticity. This study demonstrates that cofilin overactivation improves hippocampus-dependent short-term memory in the object-location task, in both male and female mice. Furthermore, cofilin^S3A^ overexpression did not affect anxiety-like behavior in the elevated plus maze in either sex. Interestingly, there were sex differences, as, for example, male mice performed better in the OLM task and made more entries in the EPM. Surprisingly, cofilin^S3A^ overexpression did not affect phosphorylation of hippocampal GluR1 AMPA receptors. Together, these data suggest that constitutively active cofilin has a beneficial effect on hippocampus-dependent short-term memory, which possibly resulted from increased synaptic plasticity in an AMPA receptor-independent fashion.

As cofilin plays a key role in spine dynamics, it is not surprising that cofilin activity is altered in certain neurological disorders, such as autism, aggression, Alzheimer’s disease, and sleep disorders (Shaw and Bamburg, 2017). Indeed, cofilin has been identified as an important factor in the memory impairments seen after sleep deprivation. A short period of only 5 h of sleep deprivation resulted in elevated cofilin levels, leading to spine loss in the hippocampus and eventually to hippocampus-dependent memory impairments (Havekes et al., 2016b). Furthermore, we also showed previously that expressing a dominant negative form of cofilin in the hippocampus prevents the spine loss and the memory deficits associated with sleep loss (Havekes et al., 2016b). Overactivating cofilin in the hippocampus, using the same viral approach same virus as used in the present study, impaired the formation of long-term object-location memories (Havekes et al., 2016b). Therefore, cofilin most likely has a different role in long-term memory compared to short-term memory, as low cofilin activity, and therefore spine stability, might be needed for memories to persist, high cofilin activity might be beneficial for short-term memory. Indeed, long-term memory initially requires structural reorganization in spines, ultimately leading to stable actin filaments (Hofer and Bonhoeffer, 2010). This actin stability might restrict receptors in their movements to keep their localization in a synapse (Hanley, 2014). For long-term memory this stability might be beneficial, however, short-term memory might be benefitted more by a highly dynamic environment within the dendritic spines (Hofer and Bonhoeffer, 2010). Therefore, short-term memory might rely less on stable actin filaments which require inactive or phosphorylated cofilin. Higher levels of active cofilin could lead to higher structural plasticity in actin filaments, which could facilitate trafficking of vesicle-containing receptors toward the synaptic density. Therefore, higher levels of active cofilin might be beneficial for short-term memory. Furthermore, during a brief period of time after LTP cofilin seems to be active before inactivation by higher levels of phosphorylated cofilin (Chen et al., 2007; Bosch et al., 2014). This could indicate that active cofilin is needed for the initial period of increased plasticity after LTP. In addition, a study showed that blocking actin polymerization in adult rats by latrunculin prevents the development of late-phase LTP (8 h), but leaves early-phase (30–50 min.) LTP intact (Fukazawa et al., 2003; Lamprecht and LeDoux, 2004). This suggests that the late phase of LTP is dependent on the stabilization of the actin filaments but not early-phase LTP. Importantly, all animals were sacrificed after wash out period to exclude potential confounding effects of behavior. It would, however, also be interesting to know how endogenous levels of (p-)cofilin change during OLM. Interestingly, Medina et al. (2020) investigated the LIMK-Cofilin 1-actin pathway in the context of fear learning, and found an ∼1.5× increase of p-cofilin 30 min after placing the mice back in the same context they were shocked in (context re-exposure) (Medina et al., 2020). In the current study, the mice injected with Cofilin^S3A^, in general have 6× higher cofilin levels compared to control, so we assume that it will outcompete any endogenous fluctuations as a result of learning. Altogether, even though cofilin activity is altered in various neurological disorders (Havekes et al., 2016b), elevated cofilin levels can also be beneficial, such as for short-term spatial memory.

The finding that hippocampal GluR1 AMPA receptor levels were unaffected in our study is unexpected. Previous findings show that these receptors are needed not only for long-term memory, but also for short-term memory by stabilizing spine morphology (Izquierdo et al., 1998; Lamprecht and LeDoux, 2004). Moreover, AMPA receptors and cofilin are functionally closely dependent on each other. For example, Gu et al. (2010) showed that increased active cofilin enhances AMPA receptor insertion into the post-synaptic membrane after LTP, while cofilin inhibition stopped AMPA receptor addition. However, while LTP was used to induce plasticity in that study, different mechanisms might occur during the formation and retrieval of object-location memories during the behavioral task in the present study. Furthermore, we looked at the phosphorylation status of GluR1 at Serine 831 which is the binding site for PKC and CAMKII and phosphorylation at this site facilitates LTP, and was necessary for normal conditioned reinforcement (Crombag et al., 2008; Kessels and Malinow, 2009). Even though phosphorylated GluR1 (ser831) levels and total GluR1 levels were not elevated, it would be interesting to examine if the phosphorylated fraction of GluR1 (ser831) receptors is higher specifically in the synapse. Therefore, future experiments should use subcellular fractioning to identify in which organelles, or where specifically GluR1 AMPA receptors (ser831) are potentially affected by cofilin overactivation. Furthermore, only male mice were used to examine the effect of cofilin overactivation at the molecular level, and future studies examine if hippocampal (p-) GluR1 AMPA receptor levels differ between sexes in the context of learning. In addition, it would be interesting to investigate if other phosphorylation sites are affected by cofilin overactivation such as the Ser 845 phosphorylation site, which is the binding site for PKA, and has been associated with spatial memory (Lee et al., 2003). In addition, while we examined GluR1 AMPA receptor levels, it would be interesting to also investigate if other subunits are affected. The GluA3 subunit is expressed highly in AMPA receptors in the hippocampus and are activated upon high levels of cAMP where they mediate synaptic plasticity through activation of both PKA and the GTPase Ras (Schwenk et al., 2014; Renner et al., 2017). Lastly, while these experiments focused on how high levels of cofilin affect GluR1 AMPA receptor phosphorylation, it might be interesting to study the relationship between cofilin and AMPA receptors under more natural conditions, for example in the context of learning.

The present study used both male and female mice to examine the effect of cofilin overactivation on memory at the behavioral level. The finding that female eGFP mice, in comparison to the male eGFP mice, did not learn is unexpected. Some studies have shown that learning and hippocampus-dependent memory could be enhanced during proestrus compared with in estrus (Frick and Berger-Sweeney, 2001; Paris and Frye, 2008). So one possible explanation could be that some of the females were in estrus during the experiment, which unfortunately, we did not measure during the experiment. Future studies should measure the estrous cycle of female mice before testing in order to minimize the variation that normally could occur due to the cycle. Although there were no differences in anxiety-like behavior in the elevated plus maze, we did find increased entries in the maze in males compared to female eGFP controls. Estrogens generally have a stimulating effect on activity levels (Morgan et al., 2004). Therefore, female rodents are often found to be more active than males, in particular during estrus. However, Morgan and Pfaff (2001) hypothesized that the effect of estrogens depends on the context. Estrogens lead to a generally increased arousal and this can result in increased activity in a safe environment but increased emotional reactivity or fear in a potentially dangerous environment (Morgan et al., 2004). Mice administered with estradiol benzoate made significantly fewer entries than control mice in the EPM but did not differ in time spent in open arms (Morgan and Pfaff, 2002). Moreover, estrogens have been shown to increase fear response as seen by lower entries in EPM (Morgan and Pfaff, 2001). These findings are in line with the data in the current study of the EPM. The mice were not handled or habituated before the EPM was performed. This could have led to an increased fear response in the EPM in females, which may have resulted in the decreased locomotor activity observed in females compared to males. Nonetheless, male and female eGFP mice showed no differences in the percentage of time spent in the open arms in the EPM, indicating no difference in anxiety levels.

Altogether, we demonstrate for the first time that cofilin overactivation specifically in the hippocampus could lead to improvements in short-term spatial memory. As most studies on, for example, sleep deprivation or cofilin have focused on long-term memory, it is of great relevance to investigate their effects on short-term memory. Therefore, it would be of additional value to examine if the effect observed in OLM can be translated to other short-term memory tasks. In addition, the underlying molecular mechanisms that facilitate short-term hippocampus-dependent memory is currently unknown. It will be interesting to see under which conditions cofilin overactivation in the hippocampus, generating a flux of increased synaptic plasticity, influences formation and retrieval of hippocampus-dependent memories. For example, it might be interesting to activate or inactivate cofilin in a temporal fashion by using photoactive version of Rac1, which inhibits cofilin via LIMK, in combination with optogenetics. Ultimately, future research on how molecular mechanisms benefit short-term memory could potentially contribute to new therapeutic approaches for patients that suffer from psychiatric disorders that are accompanied by short-term memory problems.

## Data availability statement

The raw data supporting the conclusions of this article will be made available by the authors, without undue reservation.

## Ethics statement

The animal study was approved by the National Central Authority for Scientific Procedures on Animals (CCD) and the Institutional Animal Care and Use Committee (IVD) of the University of Groningen. The study was conducted in accordance with the local legislation and institutional requirements.

## Author contributions

FR, RH, PM, and EV contributed to the conception and design of the study. FR, IWR, MO, IR, and EM collected the data. FR, IWR, MO, and IR performed the statistical analysis. FR wrote the first draft of the manuscript. IWR, MO, and IR wrote sections of the manuscript. All authors contributed to the manuscript revision and read and approved the submitted version.

## Supplementary material

Supplementary Figure 1 Cofilin overactivation in the dorsal and ventral hippocampus. Mice were bilaterally injected with pAAV9-CaMKIIa0.4-eGFP or pAAV9-CaMKIIa0.4-cofilin^S3*A*^-HA to express either eGFP or the catalytically active version of cofilin (cofilin^S3A^). Labeling was present in **(A)** dorsal, and **(B)** parts of the ventral hippocampus. An HA-tag antibody was used to label viral expression of cofilin^S3A^. Scale bar, 1 mm.

Supplementary Figure 2 Sex differences in short-term memory and anxiety-related behavior. Mice were injected with cofilin^S3A^ or eGFP, and trained for object-location memory and tested 1 hr after training. **(A)** Both male and female mice displayed a significant lower total exploration time across the three training sessions (Male: *F*_2,52_ = 18.232, *p* < 0.001; Female *F*_2,58_ = 57.704, *p* < 0.001), that did not differ between eGFP and Cofilin^S3A^ injected mice (Male: *F*_2,52_ = 1.109, *p* = 0.338; Female *F*_2,58_ = 0.021, *p* = 0.979). **(B)** Mice injected with cofilin^S3A^ performed better than eGFP injected mice in both male (*F*_1,26_ = 3.768, *p* = 0.063) and female mice (Test Statistic_1, 29_ = 175.00, *p* < 0.05). All groups, except eGFP-injected female mice explored the moved object significantly more compared to chance level (eGFP male, t_1, 13_ = 2.679, *p* < 0.05; Cofilin^S3A^, t_1, 13_ = 5.134, *p* < 0.001; eGFP female t_1, 15_ = −0.284, *p* = 0.781; Cofilin^S3A^ female *t* = 3.149, *p* < 0.01). **(C)** Percentage of time spent in the open arms of the elevated plus maze. There were no differences in percentage of time spent in open arms between cofilin^S3A^ or eGFP injected mice for both male and female mice (Male: *F*_1,16_ = 1.666, *p* = 0.215; Female *F*_1,19_ = 0.338, *p* = 0.568). **(D)** There was also no difference in the amount of entries made between cofilin^S3A^ and eGFP injected mice (Male: *F*_1,16_ = 0.578, *p* = 0.458; Female *F*_1,19_ = 0.065, *p* = 0.802). However, male mice in general made more entries compared with female mice (*F*_1,35_ = 7.029, *p* < 0.05). Data are presented as the mean, the area (band) around the mean represents the SEM, the smoothed density curve (bean) indicates the full data distribution, dots show the individual data points. **(A,B)**: eGFP, *n* = 30 (male: *n* = 14), cofilin^S3A^, *n* = 29 (male: *n* = 14). **(C,D)**: eGFP, *n* = 19 (male: *n* = 9), cofilin^S3A^, *n* = 20 (male: *n* = 9). *Indicates *p* < 0.05; # indicates *p* = 0.063.

Supplementary Figure 3 Original blots for cofilin overactivation on phosphorylation or total levels of GluR1 AMPA receptors. **(A)** Hippocampal phosphorylated GluR1 (S831) protein levels from eGFP and Cofilin^S3A^ expressing mice, with **(B)** respective GAPDH control. **(C)** Total GluR1 levels, with **(D)** respective GAPDH control. S3A: Cofilin^S3*A*^-expressing mice.
