# Supplementary figures and images for: Cofilin overactivation improves hippocampus-dependent short-term memory
Source: Front Behav Neurosci. 2023 Aug 10;17:1243524. doi: 10.3389/fnbeh.2023.1243524 (PMC10448394; doi:10.3389/fnbeh.2023.1243524)

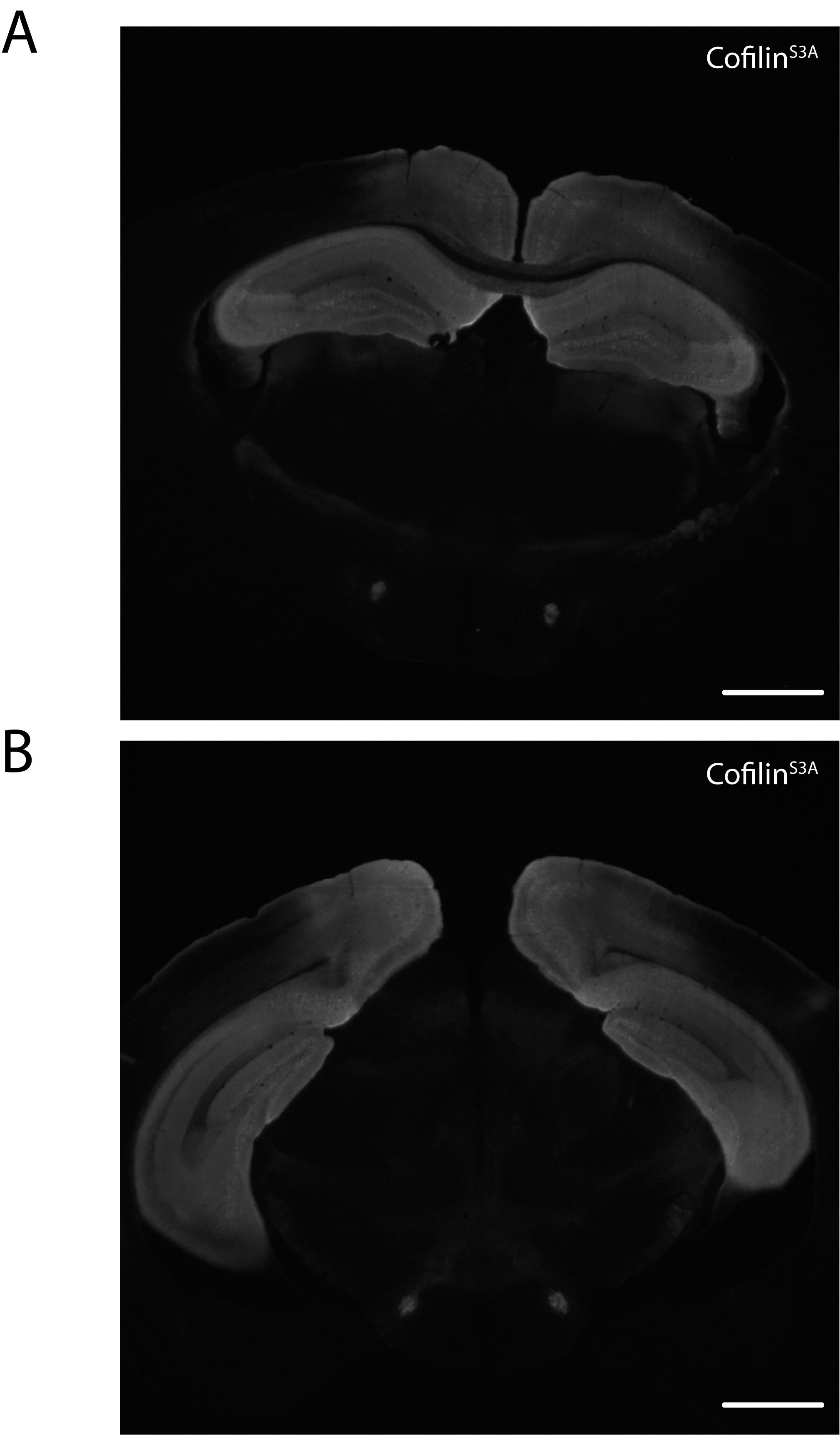

Supplement: Supplementary Figure 1 — Cofilin overactivation in the dorsal and ventral hippocampus. Mice were bilaterally injected with pAAV9-CaMKIIa0.4-eGFP or pAAV9-CaMKIIa0.4-cofilinS3A-HA to express either eGFP or the catalytically active version of cofilin (cofilinS3A). Labeling was present in (A) dorsal, and (B) parts of the ventral hippocampus. An HA-tag antibody was used to label viral expression of cofilinS3A. Scale bar, 1 mm. [file Image_1.JPEG]

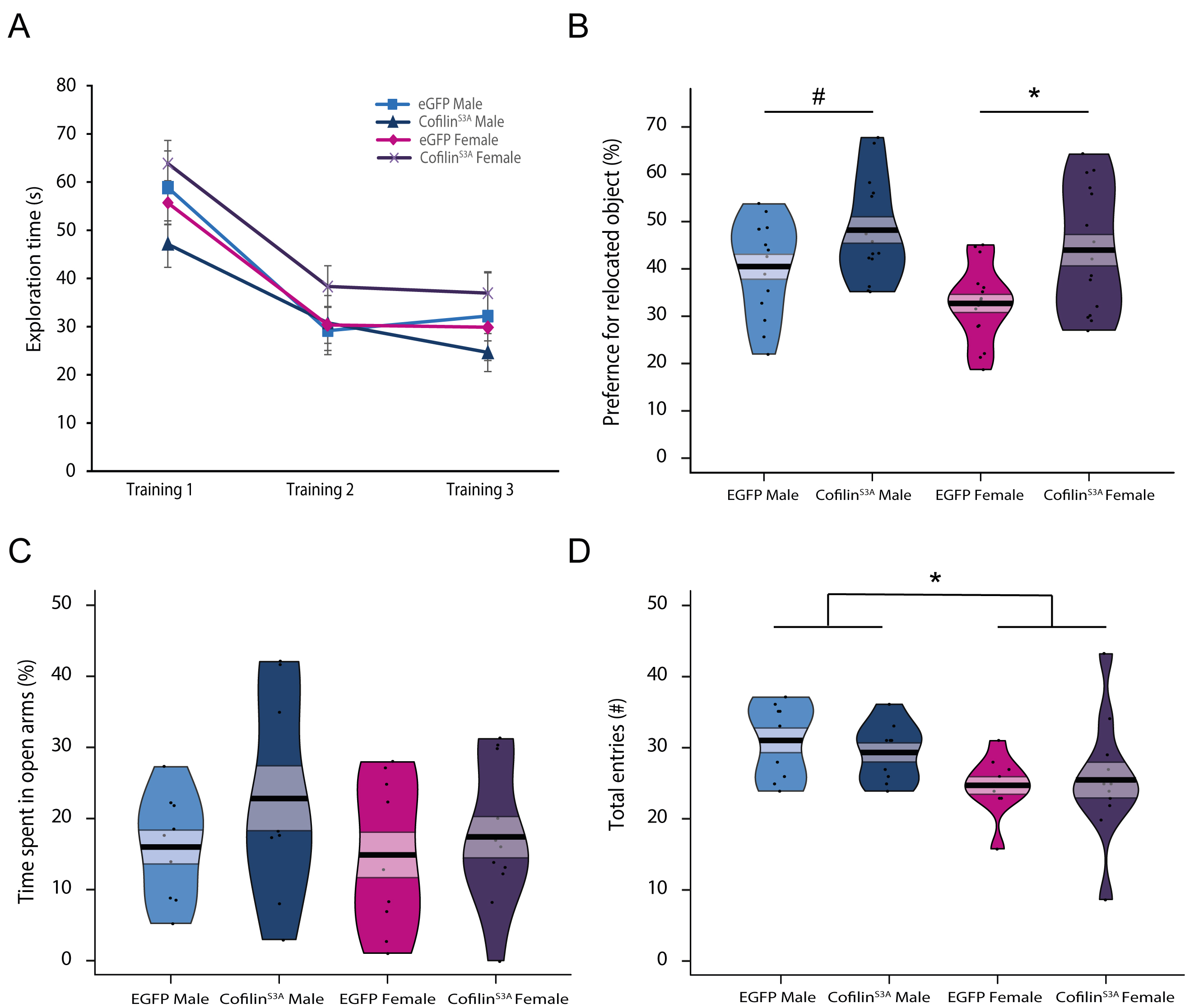

Supplement: Supplementary Figure 2 — Sex differences in short-term memory and anxiety-related behavior. Mice were injected with cofilinS3A or eGFP, and trained for object-location memory and tested 1 hr after training. (A) Both male and female mice displayed a significant lower total exploration time across the three training sessions (Male: F2,52 = 18.232, p < 0.001; Female F2,58 = 57.704, p < 0.001), that did not differ between eGFP and CofilinS3A injected mice (Male: F2,52 = 1.109, p = 0.338; Female F2,58 = 0.021, p = 0.979). (B) Mice injected with cofilinS3A performed better than eGFP injected mice in both male (F1,26 = 3.768, p = 0.063) and female mice (Test Statistic1, 29 = 175.00, p < 0.05). All groups, except eGFP-injected female mice explored the moved object significantly more compared to chance level (eGFP male, t1, 13 = 2.679, p < 0.05; CofilinS3A, t1, 13 = 5.134, p < 0.001; eGFP female t1, 15 = −0.284, p = 0.781; CofilinS3A female t = 3.149, p < 0.01). (C) Percentage of time spent in the open arms of the elevated plus maze. There were no differences in percentage of time spent in open arms between cofilinS3A or eGFP injected mice for both male and female mice (Male: F1,16 = 1.666, p = 0.215; Female F1,19 = 0.338, p = 0.568). (D) There was also no difference in the amount of entries made between cofilinS3A and eGFP injected mice (Male: F1,16 = 0.578, p = 0.458; Female F1,19 = 0.065, p = 0.802). However, male mice in general made more entries compared with female mice (F1,35 = 7.029, p < 0.05). Data are presented as the mean, the area (band) around the mean represents the SEM, the smoothed density curve (bean) indicates the full data distribution, dots show the individual data points. (A,B): eGFP, n = 30 (male: n = 14), cofilinS3A, n = 29 (male: n = 14). (C,D): eGFP, n = 19 (male: n = 9), cofilinS3A, n = 20 (male: n = 9). *Indicates p < 0.05; # indicates p = 0.063. [file Image_2.JPEG]

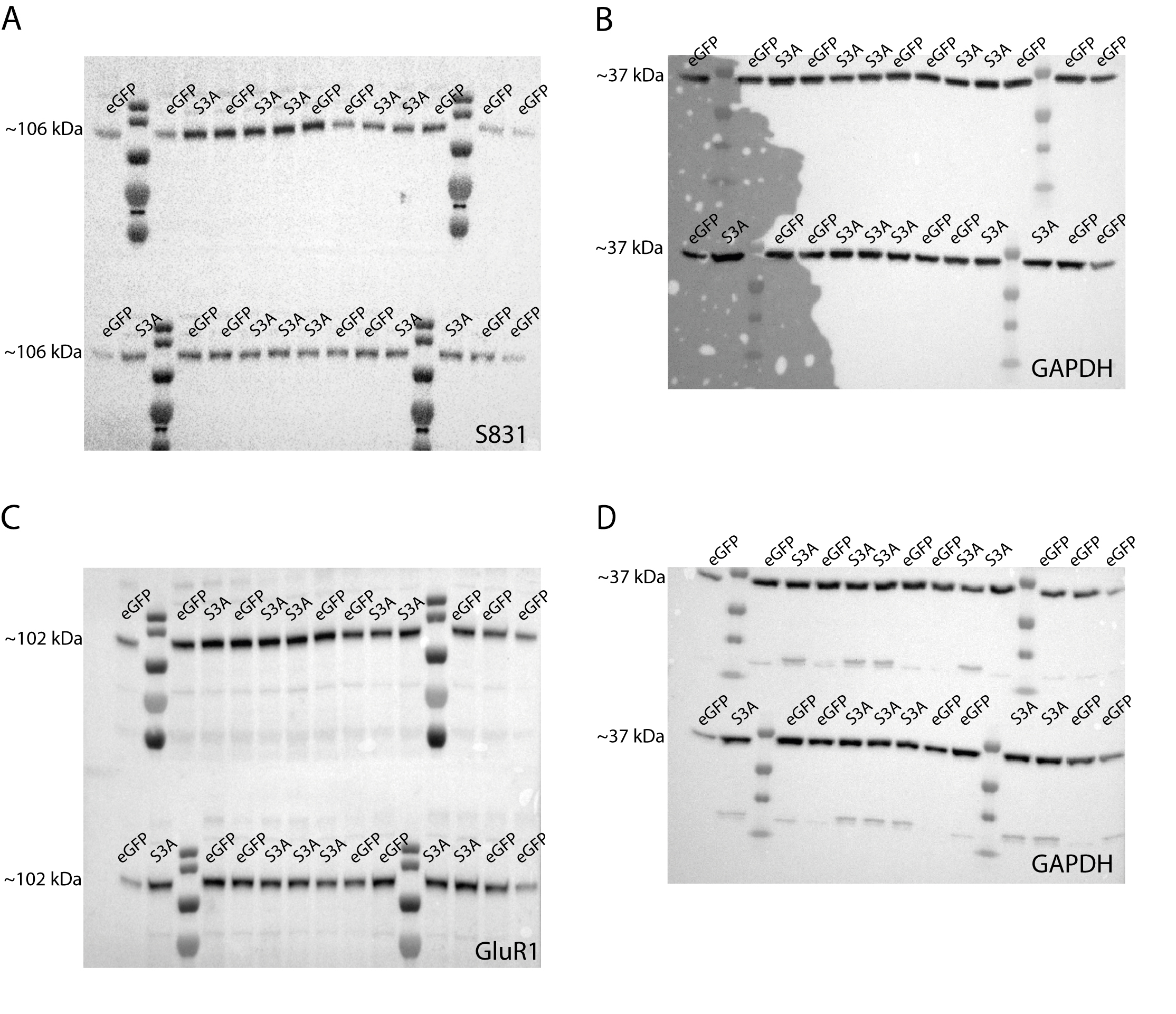

Supplement: Supplementary Figure 3 — Original blots for cofilin overactivation on phosphorylation or total levels of GluR1 AMPA receptors. (A) Hippocampal phosphorylated GluR1 (S831) protein levels from eGFP and CofilinS3A expressing mice, with (B) respective GAPDH control. (C) Total GluR1 levels, with (D) respective GAPDH control. S3A: CofilinS3A-expressing mice. [file Image_3.JPEG]
